# The Short-term Impact of Standardised Packaging on Smoking and Snus Use in Norway

**DOI:** 10.1093/ntr/ntab194

**Published:** 2021-09-24

**Authors:** Torleif Halkjelsvik, Antonio Gasparrini, Tord Finne Vedøy

**Affiliations:** 1 Department of Alcohol, Tobacco and Drugs, Norwegian Institute of Public Health, PO Box 222 Skøyen, 0213 Oslo, Norway; 2 Centre for Evaluation of Public Health Measures, Norwegian Institute of Public Health, PO Box 222 Skøyen, 0213 Oslo, Norway; 3 Department of Public Health Environments and Society, London School of Hygiene & Tropical Medicine, London, UK; 4 Centre for Statistical Methodology, London School of Hygiene & Tropical Medicine, London, UK

## Abstract

**Introduction:**

Standardised packaging on tobacco products was required in Norway July 1, 2018. We report pre-registered analyses of the potential impact on daily smoking and on daily snus use among women and men.

**Methods:**

Interrupted time series (segmented regression) on repeated cross-sectional surveys (2012–2019) from two sources: probability samples (Registry Sample, *N* = 46,957) and market research samples (Market Research Sample, *N* = 64,465) of Norwegian adults aged 16–79. Self-reported daily smoking and snus use were regressed on a step change impact variable, controlled for trend and demographics (sex, age, region, and education based on national registers in the Registry Sample, and self-reported in the Market Research Sample).

**Results:**

There were tendencies of a decline in smoking (Odds Ratio [OR] = 0.94; 95% confidence interval [CI] = 0.87, 1.02; lower-tail *p*-value [*P*_lower_] = 0.07), and women’s snus use (OR = 0.89; CI = 0.77, 1.03; *P*_lower_ = 0.06), but not men’s snus use (OR = 1.01; CI = 0.92, 1.11; *P*_lower_ = 0.59). Analyses using only the Registry Sample did not detect declines in smoking (OR = 0.99; CI = 0.88, 1.11; *P*_lower_ = 0.43) or women’s snus use (OR = 0.99; CI = 0.80, 1.24]; *P*_lower_ = 0.48), and indicated no decline in men’s snus use (OR = 1.18; CI = 1.03, 1.35; *P*_lower_ = 0.99). Exploratory analyses suggested potential acceleration of the declining trend in smoking (change in trends, OR = 0.97) and of the increasing trend in men’s snus use (OR = 1.03).

**Conclusions:**

The analyses indicate that standardised packaging in Norway did not produce a decline in men’s snus use. Results are inconclusive regarding smoking and women’s snus use. Exploratory analyses indicated a decrease in smoking and an increase in men’s snus use.

**Implications:**

We could not confirm or disconfirm whether standardised packaging is an effective tobacco control measure in a Norwegian context. According to our analyses, standardized packaging may have effects on smoking prevalence and women’s snus use, but is unlikely to reduce men’s snus use. The present results may reflect higher effectiveness of standardised packaging for products with stronger health warnings. As the results varied according to samples and outcomes, the study underlines the importance of pre-registering future analyses on this topic. Future confirmatory research should test models of gradual impact of standardised packaging.

## Introduction

In 2012, Australia became the first country to implement standardised tobacco packaging. Experiences from Australia were extensively used to argue for the implementation of standardised packaging in Norway.^1^ By July 1, 2018, standardised tobacco packages were required for cigarettes, rolling tobacco, and snus sold in Norway (a 12-month transition period started July 1, 2017). In the present article, we test and estimate the potential short-term impact of standardised tobacco packaging on the prevalence of snus use and smoking.

The prevalence of daily cigarette smoking among adults (16–74 years) in Norway declined from 21 to 10 percent in the period 2009–2018. During this period the trend and level of smoking was similar for women and men. Daily use of snus increased from 6 to 12 percent in the same period; from 11 to 17 percent among men and from 2 to 7 percent among women.^2^ With regards to tobacco control, Norway has traditionally been one of the strictest countries in Europe, typically ranking among the top five countries on the Tobacco Control Scale.^3,4^ This has primary been a result of high taxes on tobacco, bans of smoking in public places and bans on advertising.

The present regulation of health warnings states that cigarettes (factory-made or hand-rolled) must have one of two text warnings (‘Smoking kills’ or ‘Smoking is very harmful for you and your surroundings’) on the most visible side of the pack and one of fourteen combined warnings (a picture and accompanying text) on the pack reverse. Smokeless tobacco products, including snus, are required to have the text warning ‘This product may be harmful and is addictive’ on the most visible pack surface^5^; while this would be the top of the packs, tobacco companies have typically included this on the base of snus packs. The design of the health warnings were carried over when standardised (plain) packaging were implemented. As most countries introduce standardized packaging and new warnings simultaneously, this provides an opportunity to study the potential impact of standardisation without other concurrent changes in the packaging.

A Cochrane review concluded that standardised packaging may reduce smoking prevalence.^6^ The review was mainly based on research using proxies for actual behaviour (e.g., appeal of packages), with the exception of an evaluation report commissioned by the Australian government,^7^ and a peer-reviewed article by the anti-tobacco organization OxyRomandie.^8^ The former report showed a 0.6 percentage point (pp) reduction in smoking prevalence that might be attributed to the introduction of plain packages, and the latter showed a similar decrease of 3.7% (approximately 0.7 pp).

The very same data were used in studies sponsored by the tobacco industry. These studies were not able to detect any effect of the policy on smoking prevalence.^9,10^ An independent reanalysis of the data (commissioned by the academic institution of the authors of the tobacco funded studies) suggested a tendency of a reduction in smoking prevalence for minors of about 0.5 percentage points (the *p*-value was not below the typical threshold of statistical significance), and the results for adults were described as ‘considerably less convincing’,^11^ p. 15). In a more recent study, a similar effect size was reported, but the authors concluded that there was no significant decline in smoking prevalence in Australia.^12^

A concern regarding evaluation reports in general, whether sponsored by the tobacco industry or the authorities, is that methodological choices (e.g. choice of time period, analytical sample, and statistical models) can bias the results in the direction desired by the researcher and the client (cf.^13,14^). The present research aimed to evaluate the potential impact of standardised packaging with analyses registered in advance of the implementation of the policy. This limits the flexibility of the researcher and requires that deviations from the pre-registered plan are properly documented and justified.

To the best of our knowledge, we present the first pre-registered evaluation of the impact of standardised packaging on tobacco use prevalence. We chose to study daily tobacco use. The prevalence of daily tobacco use has been subject to large changes, whereas the prevalence of occasional tobacco use has remained stable at approximately 10% during the last four decades.^15^ The present study appears to be the first evaluation of the implementation of standardised packaging where no other aspects of the packaging were altered, and the first published evaluation of the impact of standardised packaging on tobacco use for a smokeless tobacco product.

We test the hypothesis that daily smoking prevalence will decrease beyond trend after the implementation of standardised cigarette packaging. As women and men differ in the level and trend of snus use,^2^ we test the same hypothesis separately for men’s snus use and women’s snus use. For smoking, we do not perform separate tests for men and women, because their level and trends in daily smoking have been similar the last two decades.^15^

## Method

### Design

The design is typically referred to as (individual-level) interrupted time series or segmented regression. The main analyses employ a step-change model, which estimates a shift in the level of tobacco use from pre- to post intervention while controlling for the overall trend and other covariates. Due to the pre-registration, the present study provides a confirmative test of the impact model used in the peer reviewed study on the short-term impact of plain packaging in Australia.^8^ Note that the step change design is not a local model of the immediate effects of the policy (such as in nonparametric regression discontinuity designs), instead the model estimates the impact across the entire pre-and post-intervention periods, which allows the estimation of the average impact on the post-intervention samples. Secondary analyses included models with an additional term for a change in trend (step + slope change) and models with no shift in level but a change in trend (slope change).

### Data

We used data from computer-assisted telephone interviews by Statistics Norway and data from a web-based survey collected by an independent market research company. The first data source (Registry Sample) is based on quarterly probability samples of Norwegian inhabitants aged 16–79 from three different surveys of varying topics (Alcohol, Tobacco and Drugs; Travelling, Holidays and other topics; Tobacco Habits), in the period January 2012 to December 2019. Sample sizes ranged from 1017 to 3212 per quarter and the response rates ranged from 51% to 65%. To account for oversampling of young adults in the second quarter and selection bias, we produced survey weights with the user-written command ‘ipfraking’ ^16^ in Stata 15.0. The construction of weights were based on population data from 2018^17^ on the following variables: age by sex (12 categories), region (7 categories), and education by sex (8 categories).

The second data source (Market Research Sample) was collected from January 2015 to December 2019 by the research agency Ipsos. The sampling is a multi-step process, where the initial data collection is based on probability samples and SMS recruitment for respondents between 18 and 59 years old, and recruitment from the company’s online market research panel for respondents above 60 years old. Three to four times a year the sample is compared with the general population in terms of demographics, and quotas are made to fill cells of underrepresented demographic groups. The data were aggregated to quarters (1500–5000 respondents per quarter) to match the periodicity of the Registry Sample. For the SMS-recruitment, the response rate is typically about 7–8%, and for the web-panel it is not possible to calculate a response rate due to multiple recruitment stages and maintenance procedures (e.g., removing inactive members of the panel). We refer to the combination of the Market Research Sample and the Registry Sample as the Full Sample (see Table S1 for demographics).

### Measures

In the Registry Sample, daily smokers were those who first confirmed that they smoked (including filter cigarettes and roll-your-own tobacco), and subsequently answered ‘Daily’ to the question ‘Do you smoke daily or occasionally?’ Daily snus users were identified based on the question ‘Do you use snus daily, occasionally or never.’ In the Market Research Sample, respondents were asked ‘What is the best description of your current smoking [snus] habits?’ Daily smokers/snus users were those who answered ‘Smoke [Use snus] daily.’ See^18^ for description of the other alternatives.

Covariates were Age Group, Sex, Region, Data Source (indicators for the Market Research Sample and the three subsamples of the Registry Sample), and Education (See Table S1 for categories of covariates). In the Registry Sample, demographic data was based on national registry data linked and categorised by Statistics Norway. In the Market Research Sample, these data were self-reported.

The step change impact variable is coded 0 in the period from Q1 2012 to Q4 2017 (pre-intervention), and 1 for Q3 2018 to Q4 2019 (post-intervention). For the phase-in (defined a priori based on informal information from the industry), the same term is coded 0.33 (Q1 2018) and 0.66 (Q2 2018). Time trend is a continuous variable measuring quarters from 1 (2012 Q1) to 32 (2019 Q4).

In the analysis with slope and step change, the slope change variable is coded 0 for Q2 2018 and increases by one for each subsequent quarter (step change variable is 1 for Q2 2018 and later, 0 otherwise). In the slope change only model, the slope change variable is coded 1 in Q2 2018 and increases by one for each subsequent quarter.

### Analyses

We performed logistic regressions in Stata 15.0 using ‘logit’ for covariate-adjusted analyses on the Full Sample and ‘svy: logit’ for weighted analyses on the Registry Sample. As there was no theoretical reason for an increase in tobacco use, we reported one-sided lower-tail p-values for the pre-registered impact model (step change). For readers who prefer estimation over hypothesis testing, we also report two-sided confidence intervals. Due to baseline differences in level and trends, we performed separate regressions for women’s and men’s snus use, and estimated the average percentage point change across sex by unweighted meta-analysis in the R package ‘metafor’.^19^

To aid the interpretation of the results, we calculated Bayes Factors according to the approach described by Dienes.^20^ These calculations were not pre-registered. The priors were based on expectations from a tobacco expert panel.^21^ The alternative hypothesis (plausible range of effect sizes in terms of percentage point changes) was a half-normal distribution with mean = 0 and standard deviation (SD) = 0.40 for smoking. For snus use, we used the SD 0.25 for women and 0.70 for men (the SDs corresponded to odds ratios of approximately 0.96).

The pre-registered analysis plan (https://osf.io/fdsuy) specified the use of data from a Health Survey, but the data was not yet available, and the data would only provide a single, large data point post-intervention and two data points pre-intervention. We also excluded two subsamples from the Registry Sample (survey on Alcohol, Tobacco, and Drugs, second quarters, 2012 and 2013), because they did not include register-based education (separate education categories for these pre-intervention data would render them useless for impact estimation). These two measurement points were already covered by other Registry Sample data. In advance, we decided to exclude one pre-registered data point due to the Covid-19 pandemic.

## Results

**Figure 1.**
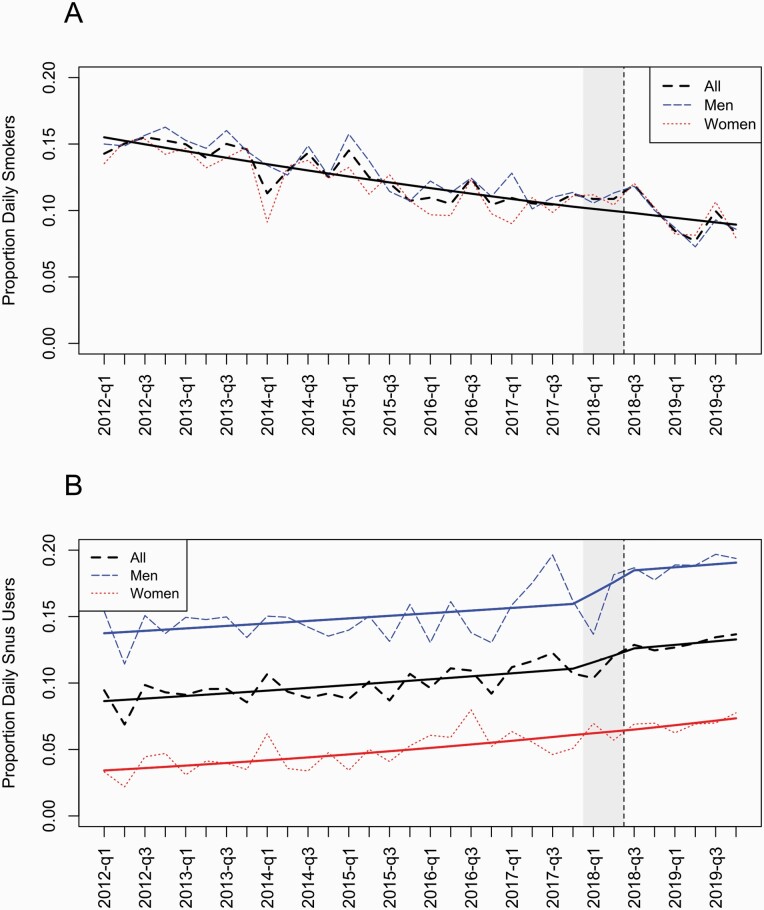
Weighted proportions of daily smokers (Panel A) and daily snus users (Panel B) in the Registry Sample before and after implementation of standardised tobacco packaging. Predicted values (solid lines) from logistic regression models. Grey shaded area indicate the phase-in and vertical line indicates full implementation.

Figure 1 shows the weighted proportions of daily smokers (panel A) and daily snus users (panel B), based on the Registry Sample. The regression lines are based on models with an overall (logit-) linear trend and the step change impact variable.

Results from regression analyses on the Full Sample and the Registry Sample are presented in Table 1 (full models in Supplemental Tables S2 and S3). The analysis of smoking prevalence in the Full Sample shows a slight decrease corresponding to half a percentage point (pp). The estimated impact on snus prevalence was positive for men and negative for women, which combined gave an effect corresponding to a decrease of 0.26 pp (lower-tail *p*-value [*P*_lower_] = 0.23, 95%CI [−0.98, 0.45]). The results on snus use appear to suggest that standardised packaging could be effective for women but not men, but an exploratory analysis on the smoking data showed that the coefficient for women was slightly higher than the original pooled analysis (OR = .96 versus .94).

**Table 1.** Odds Ratios from regression analyses of the potential impact of standardised packaging with estimated counterfactual change (in percentage points) for the post-implementation period.

	N	OR [95% CI]	*p* ^a^	pp [95% CI]^b^	[Rob. 95% CI]
Full Sample					
Smoking	111411	0.94 [0.87, 1.02]	0.07	−0.47 [−1.12, 0.18]	[−2.67, 1.73]
Snus Use (Men)	55657	1.01 [0.92, 1.11]	0.59	0.13 [−1.03, 1.28]	[−1.35, 1.60]
Snus Use (Women)	54526	0.89 [0.77, 1.03]	0.06	−0.66 [−1.48, 0.17]	[−1.87, 0.56]
Registry Sample					
Smoking	46957	0.99 [0.88, 1.11]	0.43	−0.10 [−1.09, 0.90]	[−1.27, 1.08]
Snus Use (Men)	23791	1.18 [1.03, 1.35]	0.99	2.28 [0.37, 4.20]	[0.15, 4.42]
Snus Use (Women)	23102	0.99 [0.80, 1.24]	0.48	−0.03 [−1.17, 1.11]	[−1.17, 1.11]

*Note.* Covariates for the analyses of the Full Sample: Time (trend), Age, Sex, Region, Education and Data Source. Covariates for Registry Sample: Time (Trend), Data Source. Abbreviations: OR = Odds ratio; CI = Confidence Interval; pp = Percentage points; Rob. = CIs based on cluster-robust standard errors (clustered on quarters/time).

^a^Lower-tailed *p*-values from *z*-tests.

^b^Estimated Change is based on predictive margins (difference in prevalence predicted at impact = 0 and impact = 1 for the weighted post-implementation sample). At impact = 0 the prevalence were estimated as 9.7% for smoking, 16.5% for men’s snus use, and 6.9% for women’s snus in the Full Sample; and correspondingly 9.5%, 15.4% and 5.5% in the Registry Sample.

The direction of the effects were replicated in the weighted analyses on the Registry Sample (see lower panel of Table 1). However, the estimated reductions in smoking were attenuated. This was also the case for women’s snus use, and the slight increase in men’s snus use became more pronounced in the Registry Sample. Combined, the results for women’s and men’s snus use in the Registry Sample suggested an effect corresponding to an increase of 1.13 pp (*p*_lower_ = 0.97, 95%CI[−0.01, 2.26]).

In addition to the analyses reported in Table 1, we also applied an unweighted model-based approach to the Registry Sample, where we included all weighting variables (including age and age squared as continuous variables) and their interactions with trend and impact. This yielded an estimated impact of −0.13 pp (95%CI[−1.10, 0.83]) for smoking, −0.53 pp (95%CI [−1.94, 0.88]) for women’s snus and an increase of 2.25 (95%CI [0.43, 4.07]) for men’s snus use.

### Bayes Factors

The step-change analysis of the Full Sample gave a Bayes Factor (BF) of 2.02 for smoking, 1.99 for women’s snus use, and 0.60 for men’s snus use. This indicated insensitive data with anecdotal support for a decline in smoking and women’s snus use, and anecdotal support for the null in terms of men’s snus use.

The step-change analysis of the Registry Sample gave a BF of 0.86 for smoking, and 0.93 for female snus use, suggesting insensitive data. For men’s snus use, the BF was 0.36, suggesting that the null hypothesis is almost three times more likely than the alternative hypothesis.

### Sensitivity Analyses

One assumption of the above analyses is that there is no correlation between successive measurements. Such autocorrelation was not detected in partial autocorrelation plots (Supplemental Figure S1). Another assumption is that the observations are independent; in particular, the variation between quarters (after accounting for covariates) should be solely due to sampling error (i.e., no cross-sectional dependencies/contemporaneous error). The last column of Table 1 present confidence intervals that account for potential cross-sectional dependency by clustering standard errors on the time dimension. These revealed substantially widened confidence intervals for the Full Sample, but less for the Registry Sample. Further analyses using identical (covariate-adjusted) models for the Market Research Sample and the Registry Sample showed that clustering on time gave standard errors 75–200% higher than the classic standard errors in the market research data, but nearly equivalent standard errors when using the Registry Sample (cluster-robust standard error were maximum 13% higher than the classic standard errors).

Models that allowed the overall time trends to vary between the data sources gave better fit to the data and higher consistency between the results of the Full Sample and the Registry Sample for snus use (women: coefficient = 1.03, AIC change = −19; men: coefficient = 1.17, AIC change = −51). This model did not change the coefficient for smoking, and did not improve the fit of the model (AIC change = 3).

Exploratory results on occasional smoking and snus use are provided in Supplemental Table S4. The results on occasional smoking differed somewhat between the Full and the Registry Sample, with slight and statistically uncertain declines in the Full Sample across tobacco product types, but a tendency of an increase of occasional smokers in the Registry Sample (all *p*s >.08). The results are difficult to interpret because an effective tobacco control policy can both decrease and increase the number of occasional users.

Analyses that modelled the impact of the policy as both a step change and a slope change indicated slight, immediate increases in smoking and snus use, with subsequent decreases in some of the models. There are no prior results or theoretical argument that could support such a pattern, thus, to avoid possible overfitting, we constrained the supplemental models to changes in slope only. These models are presented in the lower panel of Table 2, along with sensitivity tests with shortened pre-intervention periods (see also Supplemental Figure S2, and CIs based on cluster-robust standard errors in Supplemental Table S5; model fit statistics are provided in Table S6).

**Table 2.** Odds ratios [95% CI] from Step Change Models and Slope Change Models According to Different Pre-Intervention Periods

	Pre-intervention period starts			
	2012-Q1	2013-Q1	2014-Q1	2015-Q1
	Step Change Coefficients			
Full Sample				
Smoking	0.94 [0.87, 1.02]	0.94 [0.86, 1.02]	0.92 [0.84, 1.01]	0.92 [0.83, 1.02]
Men’s Snus Use	1.01[0.92, 1.11]	1.03 [0.94, 1.14]	1.06 [0.95, 1.18]	1.12 [1.00, 1.25]
Women’s Snus Use	0.89 [0.77, 1.03]	0.92 [0.79, 1.07]	0.98 [0.83, 1.15]	1.09 [0.92, 1.29]
Registry Sample				
Smoking	0.99 [0.88, 1.11]	1.00 [0.88, 1.14]	0.98 [0.85, 1.12]	1.02 [0.86, 1.22]
Snus Use (Men)	1.18 [1.03, 1.35]	1.16 [1.00, 1.35]	1.12 [0.95, 1.31]	1.04 [0.85, 1.26]
Snus Use (Women)	0.99 [0.80, 1.24]	0.97 [0.77, 1.23]	1.01 [0.78, 1.31]	1.05 [0.77, 1.42]
	Slope Change Coefficients			
Full Sample				
Smoking	0.94 [0.92, 0.95]	0.93 [0.92, 0.95]	0.93 [0.91, 0.94]	0.92 [0.90, 0.94]
Snus Use (Men)	0.99 [0.97, 1.00]	0.99 [0.97, 1.01]	0.99 [0.97, 1.01]	1.00 [0.98, 1.02]
Snus Use (Women)	0.95 [0.92, 0.98]	0.95 [0.92, 0.98]	0.96 [0.93, 0.99]	0.97 [0.94, 1.00]
Registry Sample				
Smoking	0.97 [0.95, 1.00]	0.97 [0.94, 1.00]	0.97 [0.94, 1.00]	0.97 [0.93, 1.00]
Snus Use (Men)	1.03 [1.00, 1.06]	1.03 [1.00, 1.06]	1.02 [0.99, 1.05]	1.01 [0.97, 1.05]
Snus Use (Women)	1.00 [0.96, 1.05]	1.00 [0.96, 1.05]	1.01 [0.96, 1.06]	1.02 [0.96, 1.07]

*Note.* Covariates for the analyses of the Full Sample: Time (Trend), Age, Sex, Region, Education and Data Source. Covariates for Registry Sample: Time (Trend), Data Source.

The only model that indicated a consistent decrease in prevalence across samples and across different specifications of the pre-intervention period was the slope change model for smoking (but also note the slope change model for women’s snus use when using the Full Sample, Table 2 and Supplemental Table S5). For this model, we ran placebo intervention tests using the Registry Sample, with the implementation of the intervention displaced to all quarters between Q2 2015 and Q1 2019. These analyses showed that the decrease in slope was stronger around the time of the intervention than in the period before the intervention, but became even stronger later in the post-intervention period (see Supplemental Figure S3). The explorative slope change model obtained a Bayes Factor of 3.62 for smoking, 0.51 for female snus use, and 0.10 for male snus use.

## Discussion

In pre-registered analyses of the potential short-term impact of standardised packaging on daily tobacco use in Norway, we observed no clear decrease beyond trends in the prevalence of tobacco use. The results on smoking prevalence and on women’s snus use were inconclusive and there was no decline in men’s snus use. In exploratory analyses of alternative impact models, a negative change in slope (decline) was observed for smoking, but a positive change in slope (increase) was observed for men’s snus use. Due to excess variation (cross-sectional dependencies) and concerns about the low response rate and non-random recruitment in the Market Research Data, we focus the below discussion on results from the Registry Sample.

The results on smoking were not inconsistent with the decrease of about half a percentage point reported for the Australian data.^7,8,12^ The lower bound of the confidence interval corresponded to a decrease of one percentage point. The present effect was, however, estimated as only a tenth of a percentage point, which, even when taking into account the lower prevalence in the present study, is smaller than the estimate in the Australian studies (the odds ratio of 0.99 approximately correspond to a 0.1 percentage point decrease from 9.5% to 9.4% and a 0.2 percentage point decrease from 17.7% to 17.5%). Similarly, the analyses of women’s snus use were not inconsistent with an underlying decline beyond trend, given the uncertainty of the results, but the effect was estimated as zero. The largest estimated change was observed for male respondents, but in the opposite direction of what we had expected.

After observing the results, it is tempting to argue that we cannot expect a decrease in prevalence within 18 months. However, a study of beliefs among tobacco experts, assessed before data on the impact of standardised packaging were available, showed that several experts believed that plain packaging could have an impact on smoking prevalence within two years. The median judgment was a one percentage point decrease in prevalence from baselines between 17 and 21 percentages.^21^ This corresponds to a crude odds ratio of about 0.95–0.96, which approximates a decrease of 0.4–0.5 pp in the present sample of smokers (estimated counter-factual prevalence of about 9% post-intervention), 0.7 in the sample of male snus users (prevalence of about 16%) and 0.3 in the sample of female snus users (prevalence of about 7%).

According to our calculation of Bayes Ratios (based on the expectations above), declines in overall smoking and women’s snus use were not more likely than a null effect, and the null was nearly three times more likely than our alternative hypothesis for men’s snus use. In the exploratory slope change model, there was stronger support for a decline than no effect in the analyses of smoking, insensitive data for women’s snus use, and strong support for no decline in men’s snus use.

The potential acceleration of the decline in smoking and the increase in snus use begs the question of whether tobacco users have switched from smoking to snus use. We are not able to investigate this with the current data. In any case, if this was an effect caused by the policy, it would require a peculiar mechanism, because both products were standardized at the same time and because the increase in snus use was observable for men only. Given the effort by the tobacco industry to develop feminine tobacco packages that are particularly attractive to female users,^22^ it would be not be surprising if some demographic groups of women differed from men in their reactions to dark green military-like standardised packages. Female snus users may differ from female smokers in their aesthetic preferences or in their sensitivity to societal trends or communicative cues (considering standardised packaging as a type of risk communication). Such differences could provide a speculative mechanism for the complex results.

It is interesting that the least promising effect was observed for snus, a product with modest text-only health warnings. Although standardised tobacco packaging can alter preferences irrespective of health warnings,^23^ interactions between packaging effects may be important (e.g., ^24,25^). In a comparison between the UK and Norway in responses to tobacco packaging before and after the implementation of standardised packaging, ratings on proxy measures for quitting increased in the UK but not Norway. The authors of the study attributed this to the changed health warnings in the UK and the unchanged health warnings in Norway.^26^

## Limitations

The analyses on the Registry Sample appeared to be more reliable than analyses utilizing the entire sample (e.g., wider robust confidence intervals for the Full Sample than the Registry Sample alone). One could therefore argue that it would be best to exclude the results from the Market Research Sample. However, as we did not pre-register criteria for selecting one sample over the other, this could be interpreted as a flexible choice to adjust results in one direction or the other. We therefore chose to report all pre-registered analyses but to focus on the higher quality Registry Sample in the sensitivity analyses. Even if the Registry Sample achieved a relatively high response rate, the data is still vulnerable to selection bias. Results may also be sensitive to the mode of administration. Telephone interviews (Registry Sample) do not necessarily produce more reliable results in comparison with online surveys.^27^

A good reason for not dismissing the Market Research Sample is that the explorative slope-only model fit the data well. The cluster-robust standard errors did not diverge substantially from the classic standard errors (Table S5) and the AICs showed a better fit than the level change model (Table S6). Although several of the past analyses on the impact of standardised packaging have used step change impact models (e.g.,^8,11,28^) one could argue that our slope change model is the more appropriate (see^7,28,29^). However, as the only difference between models is the coding of the impact term, an increase in model fit also implies an increase in the estimated effect. Choosing the better fitting explorative model would just be another way of saying that we prefer the model with the largest impact of the policy. It is still reasonable to interpret the explorative analyses as any other non-registered study. Future studies should provide more confirmative (i.e., pre-registered) tests of the slope change models. These model do require assumptions that are not needed for the simple approximation of the overall effect in the step change model (e.g., will the slope gradually return to the original trend?).

We chose daily smoking as our outcome measure. This is a coarse measure of smoking in a population. Standardised packaging may have encouraged both daily and occasional tobacco users to reduce their consumption. The developments in amount of consumption should be explored in future studies. Part of the reason for introducing standardized packaging in Norway was to encourage quitting among smokers, but the authorities emphasized the potential for preventing uptake among adolescents and young adults.^1^ As daily smoking is uncommon among young adults in Norway, this particular purpose of standardized packaging is another reason why future studies could focus on other outcomes than daily smoking—and perhaps also test effects on younger subgroups.

One potential confounder is changes in tobacco price and in income. We inspected the tobacco price and income indices and observed that the trends were almost parallel and reasonably linear in terms of yearly changes (see Supplemental Figure S5). Changes in tobacco prices and income are likely captured by the linear trend, as suggested by unreported analyses that showed high collinearity between time, income and tobacco prices (VIFs > 100 for analysis of smoking) and positive gradients of tobacco price (estimated higher use from higher prices). Note that there may be other important economic parameters that we have not accounted for (e.g., changes in affordability in vulnerable subpopulations and relative price changes).

There is also a risk of other confounding events and developments, which is why control groups are often used in evaluations of policies. However, when there is no equivalent control group, a comparison group will not necessarily make the analysis more informative. For example, in a recent analysis on tobacco sales data before and after plain packaging in Australia, the results may be driven by changes beyond trend in the control country New Zealand (see Table 3 in^30^).

The present results can only be generalised to the Norwegian population if one is willing to assume that the samples are representative or that the models are sufficiently adjusted. The results may not be generalizable to populations in countries that have higher smoking prevalence and less restrictive tobacco control policies.

A major strength of the present study is that the Norwegian context provides a relatively clean test of standardised packaging. There was no change in the health warnings co-occurring with the implementation of the policy and the tobacco taxes has grown steadily over the last years. To underscore the value of pre-registration, we note that the present analyses could have been published as strong evidence against standardised packaging by presenting data on snus use, or as evidence supporting standardised packaging by presenting the explorative slope change model for smoking.

## Conclusion

In a relatively clean test of standardised packaging, with no concurrent changes in health warnings or other major policies, we found no decline in men’s daily snus use in Norway. Results on smoking and on women’s snus use were inconclusive, however, exploratory analyses suggested that a declining trend in smoking could have been accelerated by the policy.

## Supplementary Material

A Contributorship Form detailing each author’s specific involvement with this content, as well as any supplementary data, are available online at https://academic.oup.com/ntr.

ntab194_suppl_Supplementary_Materials

ntab194_suppl_Supplementary_Taxonomy-form

## Data Availability

Data not publically available.
