# Supplementary material for: The Short-term Impact of Standardised Packaging on Smoking and Snus Use in Norway
Source: Nicotine Tob Res. 2021 Sep 24;24(2):220–6. doi: 10.1093/ntr/ntab194 (PMC8807171; doi:10.1093/ntr/ntab194)
Supplement: ntab194_suppl_Supplementary_Materials [file ntab194_suppl_supplementary_materials.docx]

**Supplemental Materials for**

**“The short-term impact of standardised packaging on smoking and snus use in Norway”**

*Supplemental Table S1. Demographics, Full Sample (Market Research Sample + Registry Sample).*

|  | | % | Missing % |
| --- | --- | --- | --- |
| Daily snus use | | 10.65 | 0.28 |
| Daily smoking | | 11.29 | 0.14 |
| Data source | |  | 0.00 |
|  | Market Research Sample | 58.02 |  |
|  | Alcohol, Tobacco and Drugs | 11.58 |  |
|  | Travelling/Omnibus | 19.51 |  |
|  | Tobacco Habits | 10.89 |  |
| Education | |  | * |
|  | Compulsory/lower/no record | 10.80 |  |
|  | Upper Secondary | 16.39 |  |
|  | Higher | 10.80 |  |
|  | Higher+ | 3.99 |  |
|  | Self-rep. Other/Undefined | 4.07 |  |
|  | Self-rep. Compulsory | 18.77 |  |
|  | Self-rep. Upper Secondary | 23.12 |  |
|  | Self-rep. Higher | 12.04 |  |
| Age | |  | 0.00 |
|  | 15-17 | 2.92 |  |
|  | 18-21 | 6.53 |  |
|  | 22-25 | 6.63 |  |
|  | 26-30 | 7.91 |  |
|  | 31-35 | 8.99 |  |
|  | 36-40 | 10.23 |  |
|  | 41-45 | 9.68 |  |
|  | 46-50 | 10.37 |  |
|  | 51-55 | 7.53 |  |
|  | 56-60 | 7.44 |  |
|  | 61-65 | 7.32 |  |
|  | 66-70 | 7.15 |  |
|  | 71-75 | 5.10 |  |
|  | 76+ | 2.21 |  |
| Geographical Region | |  | 0.21 |
|  | Oslo og Akershus | 26.47 |  |
|  | Hedmark og Oppland | 7.12 |  |
|  | Sør-Østlandet | 17.56 |  |
|  | Agder og Rogaland | 13.83 |  |
|  | Vestlandet | 17.49 |  |
|  | Trøndelag | 8.49 |  |
|  | Nord-Norge | 8.82 |  |
| Sex | |  | 0.00 |
|  | Women | 50.02 |  |
|  | Men | 49.98 |  |

Note. *Missing data are included in the categories “Compulsory/lower/no record” and “Self-rep. Other/Undefined”. Abbreviation: Self-rep. = Self-reported (Market Research Data only).

*Supplemental Table S2. Unweighted covariate-adjusted analysis on Full Sample (Market Research Sample + Registry Sample).*

|  | | Smoking | Men’s Snus Use | Women’s Snus Use |
| --- | --- | --- | --- | --- |
|  | | OR (SE) | OR (SE) | OR (SE) |
| *Constant* | | 0.020 (0.003) | 0.125 (0.014) | 0.059 (0.010) |
| Trend | | 0.983 (0.002) | 1.005 (0.003) | 1.020 (0.005) |
| Impact | | 0.944 (0.038) | 1.010 (0.046) | 0.891 (0.064) |
| Data source | |  |  |  |
|  | Market Research Sample | 1.000 | 1.000 | 1.000 |
|  | Alcohol, Tobacco and Drugs | 1.288 (0.073) | 1.179 (0.093) | 0.808 (0.106) |
|  | Travelling/Omnibus | 1.442 (0.077 | 1.061 (0.083) | 0.756 (0.099) |
|  | Tobacco Habits | 1.373 (0.077) | 1.080 (0.087) | 0.791 (0.107) |
| Education | |  |  |  |
|  | Compulsory/lower/no record | 1.000 |  |  |
|  | Upper Secondary | 0.547 (0.019) | 1.012 (0.046) | 0.926 (0.073) |
|  | Higher | 0.232 (0.011) | 0.781 (0.044) | 0.806 (0.067) |
|  | Higher+ | 0.101 (0.009) | 0.588 (0.046) | 0.600 (0.080) |
|  | Self-rep. Other/Undefined | Redundant | Redundant | Redundant |
|  | Self-rep. Compulsory | 0.785 (0.037) | 1.046 (0.073) | 0.873 (0.103) |
|  | Self-rep. Upper Secondary | 0.399 (0.019) | 0.979 (0.069) | 0.816 (0.095) |
|  | Self-rep. Higher | 0.197 (0.012) | 0.638 (0.049) | 0.501 (0.064) |
| Age | |  |  |  |
|  | 15-17 | 1.000 | 1.000 | 1.000 |
|  | 18-21 | 3.349 (0.480) | 2.456 (0.205) | 2.997 (0.346) |
|  | 22-25 | 6.585 (0.933) | 2.998 (0.256) | 3.984 (0.469) |
|  | 26-30 | 9.980 (1.385) | 2.869 (0.242) | 2.778 (0.331) |
|  | 31-35 | 11.956 (1.649) | 2.272 (0.193) | 1.412 (0.174) |
|  | 36-40 | 13.069 (1.792) | 1.953 (0.165) | 0.907 (0.116) |
|  | 41-45 | 14.837 (2.029) | 1.761 (0.151) | 0.759 (0.098) |
|  | 46-50 | 17.025 (2.315) | 1.451 (0.125) | 0.463 (0.063) |
|  | 51-55 | 16.965 (2.320) | 0.989 (0.091) | 0.278 (0.045) |
|  | 56-60 | 16.207 (2.219) | 0.589 (0.058) | 0.165 (0.031) |
|  | 61-65 | 16.048 (2.201) | 0.455 (0.047) | 0.082 (0.020) |
|  | 66-70 | 12.927 (1.782) | 0.227 (0.027) | 0.042 (0.014) |
|  | 71-75 | 10.444 (1.473) | 0.129 (0.021) | 0.024 (0.012) |
|  | 76+ | 7.352 (1.139) | 0.077 (0.022) | Omitted |
| Geographical Region | |  |  |  |
|  | Oslo og Akershus | 1.000 |  |  |
|  | Hedmark og Oppland | 1.100 (0.047) | 1.059 (0.054) | 0.873 (0.074) |
|  | Sør-Østlandet | 1.208 (0.039) | 0.925 (0.036) | 0.979 (0.057) |
|  | Agder og Rogaland | 1.125 (0.039) | 0.768 (0.032) | 0.833 (0.053) |
|  | Vestlandet | 0.997 (0.034) | 0.889 (0.034) | 0.949 (0.054) |
|  | Trøndelag | 0.921 (0.040) | 1.269 (0.057) | 1.175 (0.083) |
|  | Nord-Norge | 1.116 (0.045) | 1.171 (0.054) | 1.108 (0.078) |
| Sex | |  |  |  |
|  | Women | 1.000 |  |  |
|  | Men | 1.045 (0.022) |  |  |

*Note.* Self-reported Other/Undefined education is redundant because self-reported demographics are only used for the Market Research Sample (Data Source + the constant renders the category redundant). The oldest age category for women’s snus use was omitted by Stata due to perfect predictions. Abbreviations: OR=Odds Ratio; SE = Standard Error.

*Supplemental Table S3. Weighted Analysis on the Registry Sample*.

|  | | Smoking | Men’s Snus Use | Women’s Snus Use |
| --- | --- | --- | --- | --- |
|  | | OR (SE) | OR (SE) | OR (SE) |
| *Constant* | | 0.173 (0.009) | 0.169 (0.109) | 0.035 (0.004) |
| Trend | | 0.982 (0.002) | 1.007 (0.003) | 1.026 (0.006) |
| Impact | | 0.989 (0.059) | 1.180 (0.082) | 0.994 (0.111) |
| Data source | |  |  |  |
|  | Substance Use | 1.000 | 1.000 | 1.000 |
|  | Travelling/Omnibus | 1.090 (0.043) | 0.935 (0.042) | 0.988 (0.072) |
|  | Tobacco Habits | 1.047 (0.045) | 0.914 (0.045) | 1.004 (0.079) |

Abbreviations: OR=Odds Ratio; SE = Standard Error.

*Supplemental Table S4. Regression results on the prevalence of occasional smoking and occasional snus use: Odds Ratios (OR) and estimated counterfactual change in percentage points (pp) for the post-implementation period.*

|  | | | N | OR [95% CI] | p^a^ | pp [95% CI]^b^ | [Rob. 95% CI] |
| --- | --- | --- | --- | --- | --- | --- | --- |
| Full Sample | | |  |  |  |  |  |
|  | | Smoking | 111411 | 0.95 [0.87, 1.03] | 0.20 | -0.36 [-0.93, 0.21] | [-1.44, 0.73] |
|  | | Snus Use (Men) | 55657 | 0.94[0.82, 1.08] | 0.41 | -0.25 [-0.87, 0.36] | [-0.94, 0.44] |
|  | | Snus Use (Women) | 54526 | 0.85 [0.70, 1.02] | 0.08 | -0.44 [-0.95, 0.08] | [-1.08, 0.20] |
| Registry Sample | | |  |  |  |  |  |
|  | Smoking | | 46957 | 1.12 [0.99, 1.27] | 0.08 | 0.85 [-0.09, 1.79] | [-0.33, 2.04] |
|  | Snus Use (Men) | | 23791 | 1.01 [0.80, 1.26] | 0.95 | 0.04 [-1.08, 1.15] | [-0.85, 0.92] |
|  | Snus Use (Women) | | 23102 | 0.96 [0.71, 1.29] | 0.78 | -0.12 [-0.97, 0.72] | [-0.99, 0.74] |

*Note.* Covariates for Full Sample: Time (trend), Age, Sex, Region, Education and Data Source. Covariates for Registry Sample: Time (Trend), Data Source. Abbreviations: OR = Odds ratio; CI = Confidence Interval; pp = Percentage point change; Rob. = CIs based on cluster-robust standard errors (clustered on quarters/time).

^a^Two-tailed p-values from z-tests.

^b^Estimated Change is based on predictive margins (difference in prevalence predicted at impact = 0 and impact = 1 for the weighted post-implementation sample). At impact = 0 the prevalence were: 7.8% for smoking, 4.7% for men’s snus use, and 3.0% for women’s snus use in upper panel; lower panel: 7.7%, 5.2% and 3.0%.

*Supplemental Table S5. Odds ratios with 95% confidence intervals based on cluster-robust standard errors (clustered on time), step change and slope change models according to different pre-intervention periods.*

|  | | | Pre-intervention period starts | | | | | | | |
| --- | --- | --- | --- | --- | --- | --- | --- | --- | --- | --- |
|  | | | 2012-Q1 |  | 2013-Q1 |  | 2014-Q1 |  | 2015-Q1 |  |
|  | | | Step Change Coefficients | | | | | | | |
| Full Sample | | |  | |  | |  | |  | |
|  | | Smoking | 0.94 [0.72, 1.24] | | 0.94 [0.72, 1.23] | | 0.92 [0.70, 1.20] | | 0.92 [0.71, 1.20] | |
|  | | Men’s Snus Use | 1.01[0.90, 1.13] | | 1.03 [0.92, 1.16] | | 1.06 [0.94, 1.19] | | 1.12 [1.00, 1.25] | |
|  | | Women’s Snus Use | 0.89 [0.73, 1.09] | | 0.92 [0.74, 1.13] | | 0.98 [0.79, 1.20] | | 1.09 [0.96, 1.24] | |
| Registry Sample | | |  | |  | |  | |  | |
|  | Smoking | | 0.99 [0.86, 1.13] | | 1.00 [0.87, 1.15] | | 0.98 [0.83, 1.15] | | 1.02 [0.83, 1.26] | |
|  | Snus Use (Men) | | 1.18 [1.01, 1.38] | | 1.16 [0.99, 1.37] | | 1.12 [0.94, 1.33 | | 1.04 [0.85, 1.26] | |
|  | Snus Use (Women) | | 0.99 [0.80, 1.24] | | 0.97 [0.77, 1.24] | | 1.01 [0.76, 1.35] | | 1.05 [0.79, 1.40] | |
|  | | | Slope Change Coefficients | | | | | | | |
| Full Sample | | |  | |  | |  | |  | |
|  | Smoking | | 0.94 [0.88, 1.00] | | 0.93 [0.87, 1.00] | | 0.93 [0.87, 0.99] | | 0.92 [0.86, 0.99] | |
|  | Snus Use (Men) | | 0.99 [0.97, 1.01] | | 0.99 [0.97, 1.02] | | 0.99 [0.97, 1.02] | | 1.00 [0.97, 1.02] | |
|  | Snus Use (Women) | | 0.95 [0.91, 0.99] | | 0.95 [0.92, 0.99] | | 0.96 [0.92, 1.00] | | 0.97 [0.94, 1.00] | |
| Registry Sample | | |  | |  | |  | |  | |
|  | Smoking | | 0.97 [0.95, 1.00] | | 0.97 [0.95, 1.00] | | 0.97 [0.94, 0.99] | | 0.97 [0.93, 1.00] | |
|  | Snus Use (Men) | | 1.03 [1.00, 1.06] | | 1.03 [1.00, 1.06] | | 1.02 [0.99, 1.05] | | 1.01 [0.97, 1.05] | |
|  | Snus Use (Women) | | 1.00 [0.97, 1.04] | | 1.00 [0.97, 1.04] | | 1.01 [0.97, 1.05] | | 1.02 [0.98, 1.06] | |

*Note.* Covariates for the analyses of the Full Sample: Time (trend), Age, Sex, Region, Education and Data Source. Covariates for Registry Sample: Time (trend), Data Source.

*Supplemental Table S6. Akaike information criterion (AIC) for the main and explorative models.*

|  | | Smoking | Men’s Snus Use | Women’s Snus Use |
| --- | --- | --- | --- | --- |
| Full sample | |  |  |  |
|  | Level change | 65806.4 | 43618.8 | 21246.8 |
|  | Slope change | 65753.1 | 43617.8 | 21236.9 |
| Registry Sample | |  |  |  |
|  | Level change | 30165.7 | 18883.7 | 8076.4 |
|  | Slope change | 30148.8 | 18901.8 | 8072.2 |


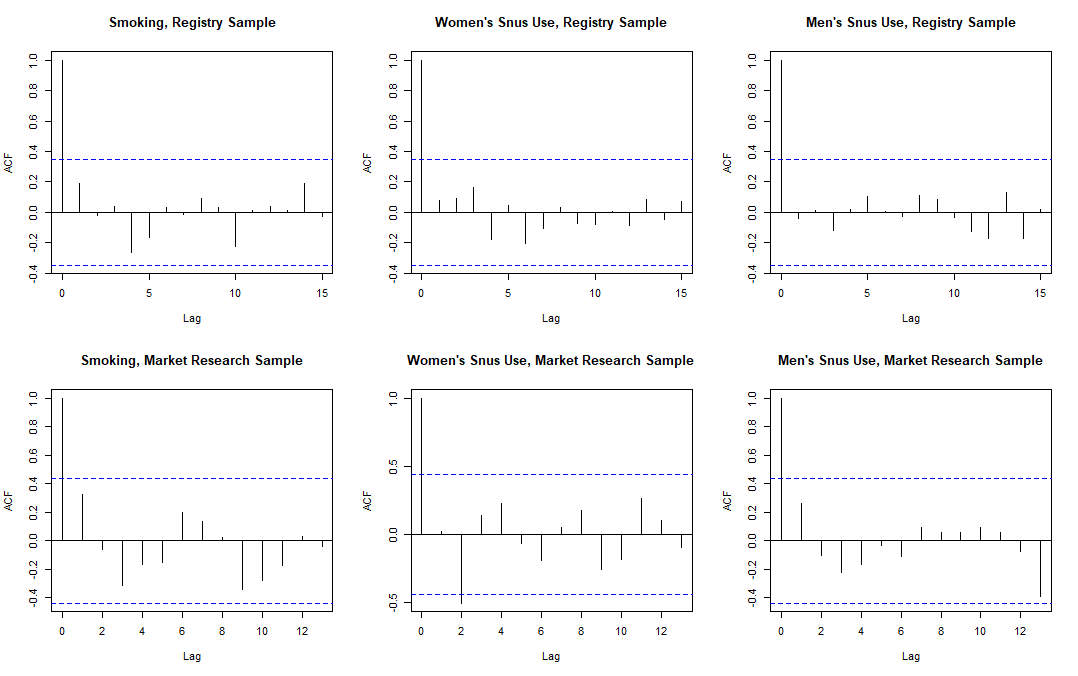


*Supplemental Figure S1.* Partial autocorrelation plots for the Registry Sample (weighted analysis, controlled for subsample source) and the Market Research Sample (unweighted analysis controlled for sex, age, education, and geographical region).


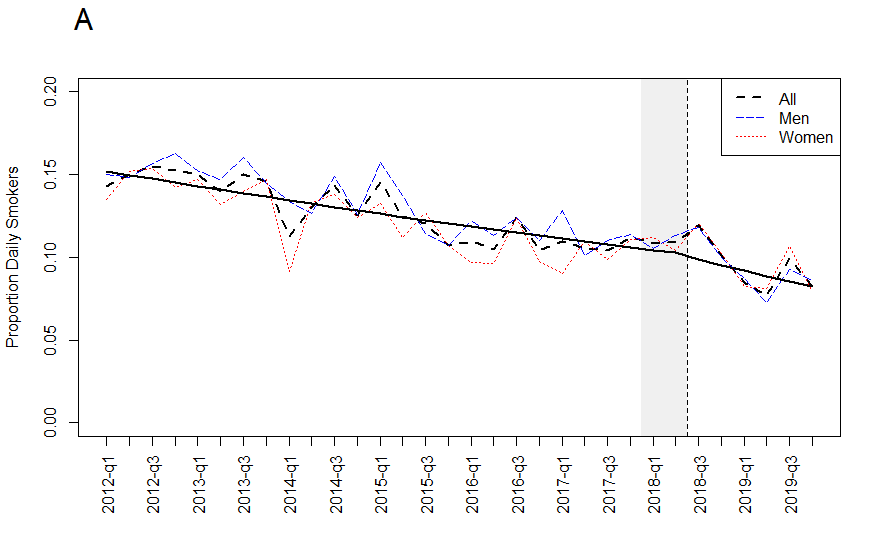


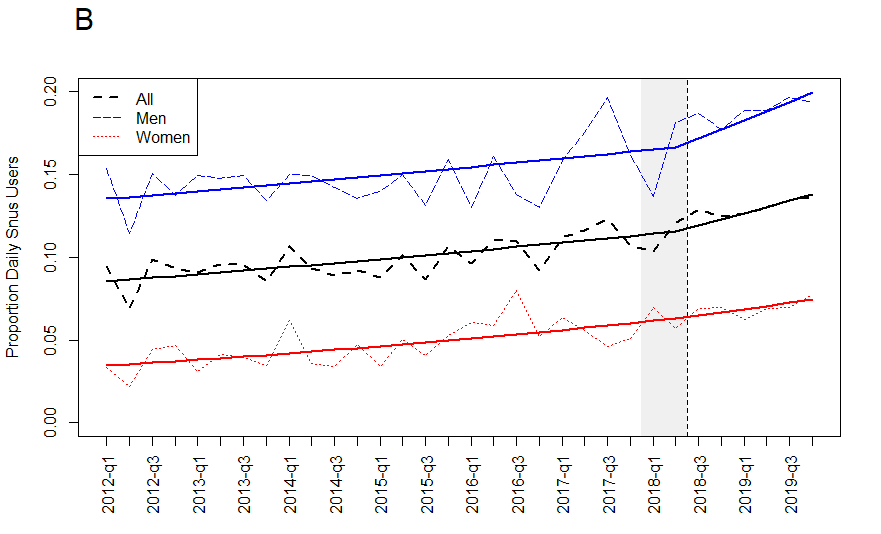


*Supplemental Figure S2.* Weighted proportions of daily smokers (Panel A) and daily snus users (Panel B). Solid lines represent predicted values from change in slope models.


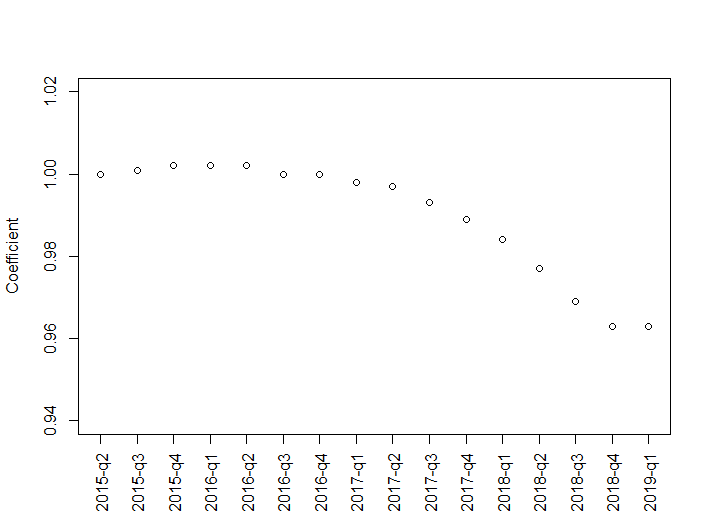


*Supplemental Figure S3.* Placebo intervention tests for the slope change model of smoking prevalence show that the decrease in the slope was stronger around the time of the intervention than in the period before the intervention but even stronger after the intervention. Although it is speculative to interpret this pattern, it could indicate an accelerating decrease triggered by the intervention. It is however somewhat strange for a policy to have increasing impact after a delay. In addition, this must be a mechanism that works selectively on smokers, as the slope change model gave an estimated *increase* in the prevalence of snus use.


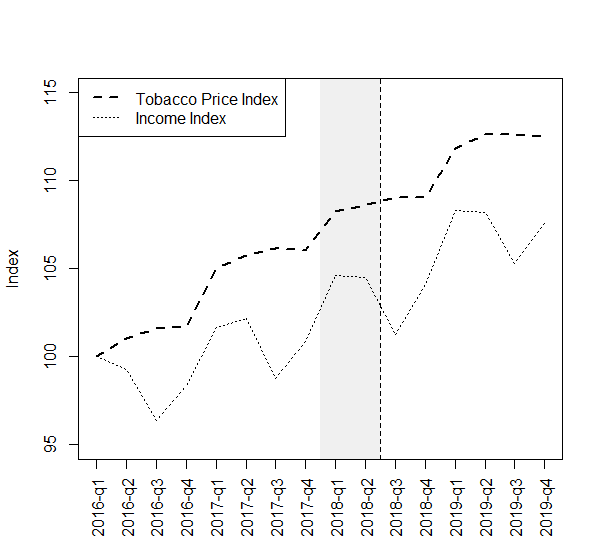


*Supplemental Figure S4*. Development in tobacco prices and in income according to Statistics Norway (Tobacco Price: <https://www.ssb.no/statbank/table/03013/> ; Income: <https://www.ssb.no/statbank/table/11654> ).
